# Case Report: Expanding the spectrum of renal involvement in adult-onset still’s disease: a case of focal proliferative glomerulonephritis and ischemic nephropathy

**DOI:** 10.3389/fmed.2025.1609256

**Published:** 2025-10-16

**Authors:** Lina Zhang, Yan Song, Suxia Wang, Shengguang Li

**Affiliations:** ^1^Department of Rheumatology and Immunology, Peking University International Hospital, Beijing, China; ^2^Department of Nephrology, The Fourth Medical Center of Chinese PLA General Hospital, Beijing, China; ^3^Electronic Microscopy Office, Peking University First Hospital, Beijing, China

**Keywords:** adult-onset still’s disease, focal proliferative glomerulonephritis, ischemic nephropathy, systemic inflammation, immunosuppressive therapy

## Abstract

Adult-onset Still’s disease (AOSD) is a rare systemic inflammatory disorder characterized by high spiking fever, evanescent rash, and arthritis. Renal involvement in AOSD is uncommon but clinically significant, with varied manifestations such as AA amyloidosis, thrombotic microangiopathy (TMA), and collapsing glomerulopathy. In this case report, we presented a unique instance of a 28-year-old female with AOSD complicated by both focal proliferative glomerulonephritis (FPGN) and ischemic nephropathy—two rare renal manifestations confirmed via renal biopsy. The patient exhibited persistent fever, joint pain, and proteinuria, which were unresponsive to initial antimicrobial treatment but showed marked improvement following corticosteroid and immunosuppressive therapy. This case expands the known spectrum of renal involvement in AOSD and underscores the importance of prompt identification and individualized therapy. We also provide a comparative analysis of reported renal pathologies in AOSD to enhance understanding and guide management strategies.

## Introduction

Adult-onset Still’s disease (AOSD) is a rare systemic autoinflammatory disorder, first described by Bywaters in 1971 as the adult counterpart of systemic-onset juvenile idiopathic arthritis (JIA), initially reported by Still in 1897 (1). AOSD is characterized by high spiking fever, evanescent salmon-colored rash, arthritis, and leukocytosis with neutrophil predominance. The pathogenesis of AOSD is believed to involve intense activation of the innate immune system, leading to the overproduction of pro-inflammatory cytokines, such as interleukin (IL)-1, IL-6, and IL-18 (2). These cytokines contribute to the systemic inflammatory response and the array of symptoms observed in AOSD patients.

Renal involvement in AOSD is relatively rare, but when present, it significantly impacts disease management and prognosis. The renal manifestations in AOSD are diverse, with AA amyloidosis being the most frequently reported complication. Other reported forms of renal involvement include thrombotic microangiopathy (TMA), collapsing glomerulopathy, and IgA nephropathy (3). Each type of renal involvement poses unique diagnostic challenges, often overlapping with other systemic complications of AOSD and requiring careful differential diagnosis. AA amyloidosis is the most common renal manifestation of AOSD, generally associated with prolonged systemic inflammation. It presents as nephrotic syndrome and, if untreated, can lead to progressive renal failure (4, 5). Thrombotic microangiopathy, characterized by endothelial damage and microvascular thrombosis, is another reported renal manifestation of AOSD. It typically presents with acute kidney injury and hematologic abnormalities, such as thrombocytopenia (6–8). Less commonly, collapsing glomerulopathy (9, 10) and IgA nephropathy (11) have also been reported, indicating that renal involvement in AOSD can present with a broad spectrum of pathologies.

In contrast, focal proliferative glomerulonephritis (FPGN) and ischemic nephropathy are exceedingly rare in AOSD. FPGN is characterized by focal or segmental proliferation of mesangial or endothelial cells, often with associated inflammatory infiltrates. Without prompt treatment, FPGN can lead to progressive renal impairment. Ischemic nephropathy results from reduced renal perfusion due to arteriolar narrowing and glomerular sclerosis, which exacerbates renal dysfunction. In a recent systematic review of 44 AOSD cases with renal involvement, neither FPGN nor ischemic nephropathy were prominent among the findings, highlighting the extreme rarity of these complications (3). To date, there have been few, if any, reported cases of AOSD complicated by both FPGN and ischemic nephropathy, making this case particularly significant.

This case report presents a unique instance of a 28-year-old female with AOSD complicated by both FPGN and ischemic nephropathy, confirmed by renal biopsy. The presentation of these two rare renal complications in a single patient expands our understanding of the spectrum of renal involvement in AOSD. In addition, this report highlights the importance of early recognition of renal complications and the need for individualized, targeted treatment strategies to improve outcomes. A comparative analysis of renal pathologies in AOSD is provided to contextualize the uniqueness of this case, offering insights that may aid clinicians in the diagnosis and management of similar cases in the future.

## Case presentation

### Clinical presentation

A 28-year-old female presented to our hospital with a five-week history of high-grade fever, sore throat, and multiple joint pain. The fever, ranging from 38 °C to 40 °C, was associated with joint pain involving the knees, wrists, and shoulders, as well as an evanescent salmon-colored rash. The patient initially received antimicrobial treatment at a local hospital, including Penicillin, Cefmetazole, and Moxifloxacin, but showed no clinical improvement. Intermittent administration of dexamethasone temporarily reduced her temperature, but the fever recurred after corticosteroid discontinuation.

Upon admission to our hospital, physical examination revealed a body temperature of 40.5 °C, blood pressure of 135/90 mmHg, and an irregularly shaped rash over the trunk and extremities. Palpable, tender lymph nodes were present in the subclavian, axillary, and groin regions bilaterally. Joint examination showed tenderness in the wrists and knees without visible swelling or deformation. Cardiopulmonary examination was unremarkable, with no abnormalities in heart or lung sounds. The liver and spleen were not palpable, but ultrasound imaging indicated a moderate amount of pericardial effusion and mild splenomegaly. Other abdominal organs, including the thyroid, liver, gallbladder, and pancreas, appeared normal on imaging. A computed tomography (CT) scan of the chest and abdomen revealed mediastinal lymph node enlargement.

### Laboratory findings and diagnosis

Laboratory findings were significant for leukocytosis, with a white blood cell (WBC) count of 19.68 × 10⁹/L and 87.6% neutrophils. Erythrocyte sedimentation rate (ESR) was markedly elevated at 98 mm/h, and high-sensitivity C-reactive protein (CRP) was 96.2 mg/L, both indicative of significant systemic inflammation. Serum ferritin was exceedingly high at > 2000 ng/mL (normal range: 11–336.2 ng/mL). Urinalysis showed proteinuria (++), with 8 to 14 red blood cells per high-power field and a 24-h urine protein level of 1.67 g. Serum creatinine was elevated at 184 μmol/L, and blood urea nitrogen (BUN) was 14.33 mmol/L, suggesting impaired renal function. Complement levels were decreased, with C3 of 0.65 g/L (normal range: 0.9–1.8 g/L) and C4 of 0.01 g/L (normal range: 0.10–0.40 g/L).

Rheumatoid factor (RF), antinuclear antibodies (ANA), and extractable nuclear antibodies (ENA) were negative, ruling out common autoimmune diseases such as systemic lupus erythematosus (SLE). Antineutrophil cytoplasmic antibody (ANCA) testing was also performed and was negative, excluding ANCA-associated vasculitis as a cause of the renal findings. Bone marrow smear suggested reactive lymphoid hyperplasia, and a biopsy of the groin lymph node confirmed reactive lymphoid hyperplasia. Screening for *Mycoplasma pneumoniae*, tuberculosis, and influenza viruses, as well as a panel of tumor markers, was negative. A PET/CT scan revealed no evidence of malignancy.

Based on the combination of persistent high fever, rash, arthritis, extreme hyperferritinemia, negative infectious and autoimmune workup, and lymph node histology showing reactive lymphoid hyperplasia, a diagnosis of adult-onset Still’s disease (AOSD) was made. The patient was started on prednisolone (15 mg three times daily), resulting in rapid defervescence within 1 week and gradual resolution of the rash.

### Renal biopsy results

Five weeks later, a follow-up evaluation revealed significant improvement. The WBC count had decreased to 9.74 × 10⁹/L, with neutrophils at 71.6%, and CRP had decreased to 46.2 mg/L. Serum ferritin was reduced to 780.0 ng/mL, indicating a response to corticosteroid therapy. Ultrasound showed reduced pericardial effusion, and lymphadenopathy was no longer detectable. However, persistent proteinuria (++, 1.46 g/24 h) and microscopic hematuria (≈10 RBC/HPF) indicated ongoing renal involvement. In light of the persistent proteinuria, a percutaneous renal biopsy was performed.

**Figure 1:**
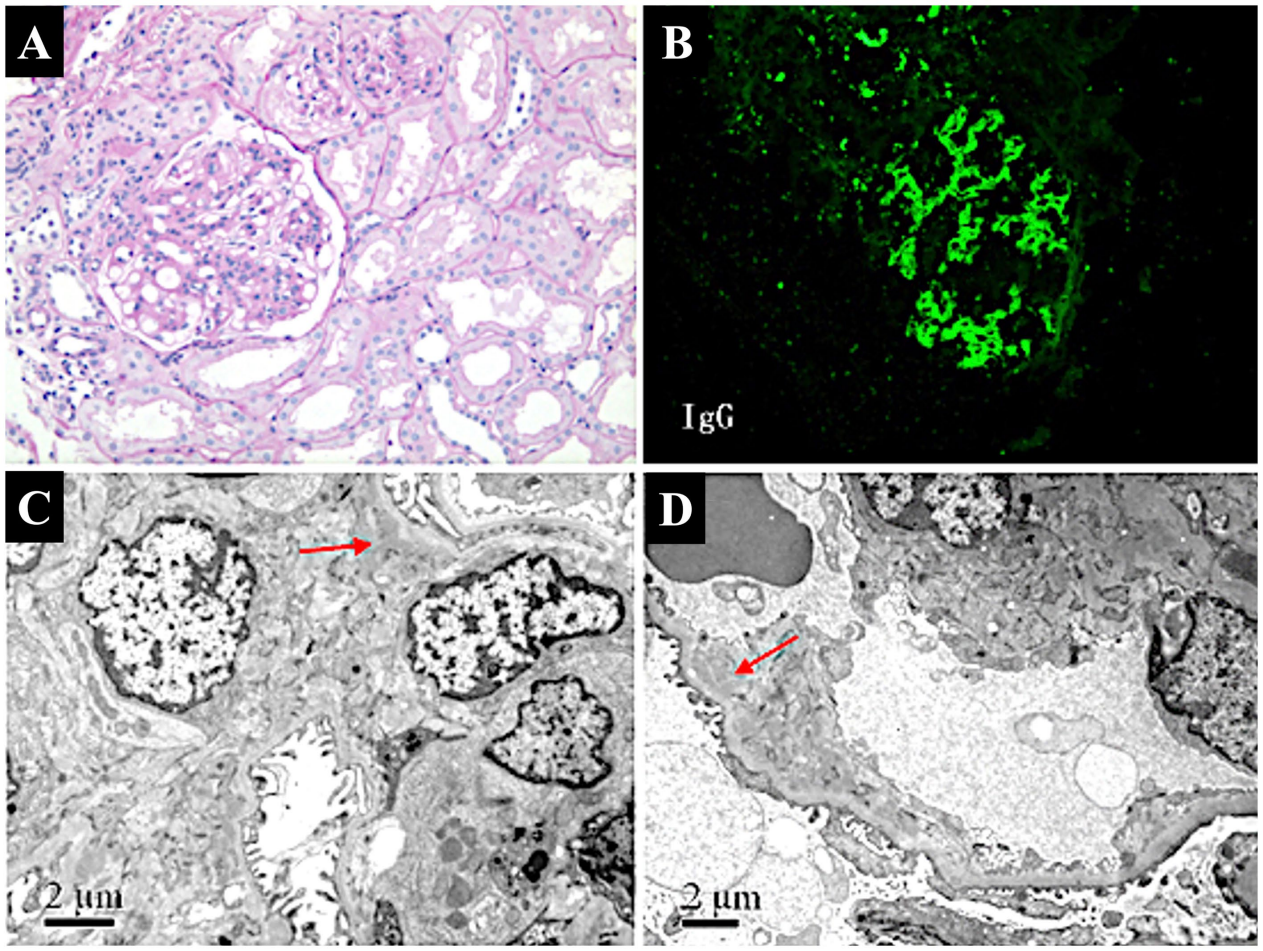
Pathological findings of renal biopsy in a patient with AOSD complicated by focal proliferative glomerulonephritis and ischemic nephropathy. **(A)** Light micrograph of focal proliferative glomerulonephritis (PAS stain, ×100). Shows moderate mesangial proliferation with segmental sclerosis, indicative of focal proliferative glomerulonephritis. **(B)** Immunofluorescence staining of renal biopsy. Demonstrates lumpy deposits of IgG in the mesangium, suggesting immune complex-mediated glomerular injury (FITC × 100). **(C)** Electron micrograph of glomerular mesangium. Reveals segmental podocyte fusion and mesangial proliferation (arrow), with mild to moderate proliferation and focal electron-dense deposits (×10,000), consistent with focal proliferative glomerulonephritis. **(D)** Electron micrograph showing ischemic changes in the glomerulus. Displays thickening of capillary walls, narrowing of the arteriolar lumen (arrow), and glomerular sclerosis (×8,000), indicative of ischemic nephropathy.

Renal biopsy (Figure 1) revealed 12 glomeruli, 4 of which showed ischemic sclerosis, while others exhibited moderate mesangial proliferation. Additional findings included shrinkage of the glomerular basement membrane, granular degeneration, and focal tubular atrophy. Focal infiltration of lymphocytes and fibrosis in the interstitium were also noted. Immunofluorescence revealed lumpy deposits of IgG, IgA, IgM, and C3 in the mesangium, while C1q was negative. Electron microscopy confirmed mild mesangial proliferation with segmental podocyte fusion, consistent with the diagnosis of focal proliferative glomerulonephritis (FPGN) complicated by ischemic nephropathy.

### Treatment and follow-up

The treatment regimen was adjusted following the biopsy results. Leflunomide 20 mg daily was added as a steroid-sparing immunosuppressant to better control the immune-mediated glomerular injury. In addition, antihypertensive therapy with Losartan and Felodipine sustained-release tablets was initiated to improve renal perfusion and mitigate further ischemic damage. Prednisolone was gradually tapered over 6 weeks to 10 mg daily. Upon re-evaluation 13 months after treatment, the patient was in good condition, with stable blood pressure at 130/90 mmHg. Laboratory results showed improved renal function, with serum creatinine at 79 μmol/L and BUN at 6.71 mmol/L. Serum ferritin remained moderately elevated at 681.5 ng/mL. Repeat urinalysis showed a 24-h urine protein level of 0.84 g, with 9 to 14 red blood cells per high-power field, indicating residual but stable renal involvement.

**Figure 2:**
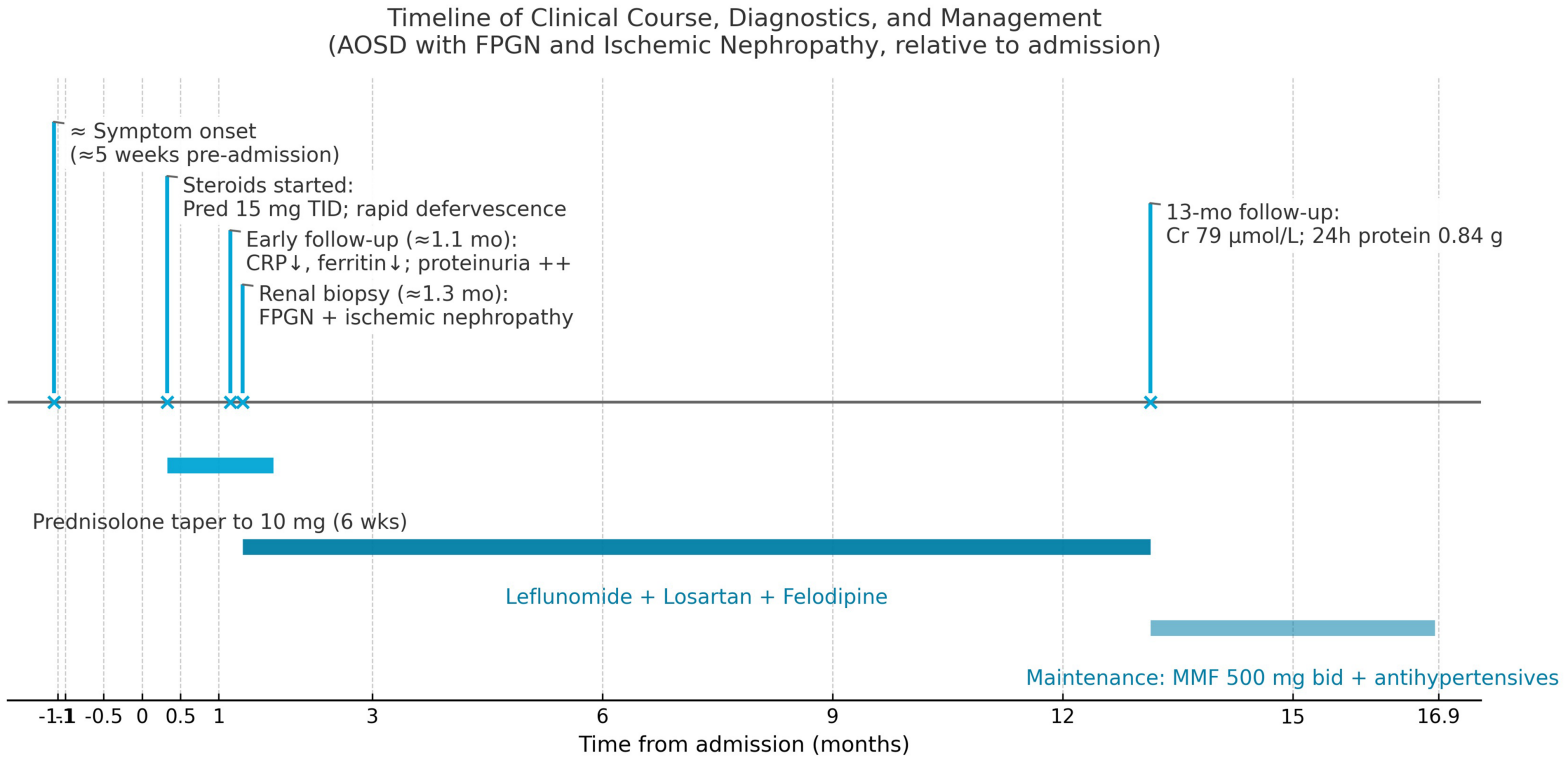
A timeline summarizing the patient’s clinical course, from initial presentation through diagnosis, treatments, and outcomes.

The patient’s maintenance regimen was further tailored to ensure lasting remission. She continued treatment with Mycophenolate mofetil (MMF) 500 mg twice daily, having transitioned to MMF for longer-term immunosuppression due to the incomplete response to leflunomide. Losartan and Felodipine were maintained for blood pressure control. Her blood pressure remained stable at 122/80 mmHg, and she remained asymptomatic during follow-up, with no further episodes of fever, joint pain, or lymphadenopathy. Her renal parameters remained stable, and proteinuria stayed low-grade. A timeline summarizing the patient’s clinical course, from initial presentation through diagnosis, treatments, and outcomes, is presented in Figure 2.

## Discussion

Renal involvement in adult-onset Still’s disease (AOSD) is a rare but clinically significant complication that can substantially impact patient outcomes. The renal pathologies associated with AOSD are diverse, with AA amyloidosis being the most frequently reported, followed by thrombotic microangiopathy (TMA), collapsing glomerulopathy, and IgA nephropathy (3). Each of these renal manifestations presents distinct clinical features and pathophysiological mechanisms, which challenges the management of AOSD. To enhance the understanding of renal pathologies in AOSD, we reviewed the literature and summarized the different types of renal involvement, including their frequencies, clinical features, and treatment approaches, in Table 1. This table provides a comprehensive overview of the various renal pathologies reported in AOSD, highlighting that focal proliferative glomerulonephritis (FPGN) combined with ischemic nephropathy was rarely documented.

**Table 1:** Comparison of renal pathology types in AOSD (3).

Renal pathology type	Frequency in systematic review	Features	Treatment approaches
AA amyloidosis	25% (11/44 cases)	Deposition of amyloid protein, often associated with chronic inflammation; presents with nephrotic syndrome, proteinuria.	Glucocorticoids, Colchicine, TNF inhibitors (e.g., Etanercept).
Thrombotic microangiopathy (TMA)	11.4% (5/44 cases)	Endothelial injury leading to microangiopathy; may present with hemolytic anemia, thrombocytopenia, and renal failure.	Glucocorticoids, Anakinra, Eculizumab, Plasma exchange.
Collapsing glomerulopathy	11.4% (5/44 cases)	Collapse of glomerular capillary tufts, often seen in association with viral infections or immune activation.	Glucocorticoids, Methotrexate, Anakinra, IVIG.
IgA nephropathy	9.1% (4/44 cases)	Mesangial deposition of IgA, often presents with hematuria and proteinuria.	Glucocorticoids, Leflunomide, NSAIDs, Azathioprine.
Minimal change disease (MCD)	6.8% (3/44 cases)	Characterized by podocyte effacement, presents primarily with nephrotic syndrome.	Glucocorticoids, Cyclosporine.
Membranous glomerulonephritis	4.5% (2/44 cases)	Thickening of glomerular capillary walls, presents with proteinuria, sometimes nephrotic syndrome.	Infliximab, Losartan.
Focal proliferative glomerulonephritis (FPGN)	Not reported in the systematic review	Focal or segmental proliferation of mesangial/endothelial cells; can lead to renal dysfunction if untreated.	Glucocorticoids, Leflunomide, Mycophenolate Mofetil (MMF).
Ischemic nephropathy	Not reported in the systematic review	Reduced renal perfusion due to arteriolar narrowing and glomerular sclerosis; leads to ischemic damage.	Blood pressure control (e.g., Losartan), Glucocorticoids.

In this case report, we described a unique instance of AOSD with a combination of FPGN and ischemic nephropathy—an association that has not been previously reported. The coexistence of these two renal pathologies offered new insights into the spectrum of renal involvement in AOSD. A comparative analysis with previously documented cases revealed that FPGN and ischemic nephropathy are rare manifestations of renal involvement in AOSD, which differed from the more frequently reported AA amyloidosis (4, 5).

FPGN is characterized by focal or segmental proliferation of mesangial or endothelial cells, often accompanied by inflammatory infiltrates, which can lead to progressive renal impairment if untreated (12). In our patient, renal biopsy revealed mesangial proliferation, basement membrane shrinkage, and focal tubular atrophy, consistent with FPGN. Immunofluorescence showed lumpy deposits of IgG, IgA, IgM, and C3 in the mesangium, and electron microscopy confirmed segmental podocyte fusion. These findings suggest an immune-mediated etiology, as FPGN in AOSD may result from immune complex deposition, leading to localized glomerular injury and activation of inflammatory pathways (13, 14).

Furthermore, ischemic nephropathy manifested in our patient as glomerular sclerosis and arteriolar narrowing, leading to reduced renal perfusion and ischemic damage. Such changes are likely secondary to chronic inflammation in AOSD, contributing to microvascular dysfunction. The systemic inflammatory response in AOSD, characterized by elevated pro-inflammatory cytokines including IL-1, IL-6, and IL-18, can lead to endothelial activation and dysfunction (15). This inflammatory milieu promotes vascular permeability, leukocyte adhesion, and thrombotic microangiopathy (TMA), ultimately resulting in impaired tissue perfusion and ischemic damage (15, 16). These events are characterized by endothelial damage, which can manifest as reduced renal perfusion, arteriolar narrowing, and subsequent ischemic damage, precisely as observed in our patient’s renal biopsy showing ischemic nephropathy. Reduced renal perfusion and subsequent ischemia can exacerbate renal dysfunction, underscoring the importance of early detection and management of renal vascular changes in AOSD patients (17, 18).

The treatment of renal involvement in AOSD is challenging and often requires a combination of immunosuppressive agents tailored to the specific renal pathology. In our patient, initial corticosteroid therapy led to partial improvement, reducing fever and systemic inflammation markers. However, persistent proteinuria necessitated further immunosuppressive therapy, including leflunomide and subsequently mycophenolate mofetil (MMF), due to incomplete response. MMF has been successfully used in other immune-mediated glomerulopathies, providing additional support for its use in AOSD with renal involvement (9, 19). Blood pressure control was also essential in managing ischemic nephropathy. The use of losartan and felodipine helped maintain adequate renal perfusion and prevent further ischemic injury, which is critical for minimizing the progression of chronic kidney disease. These agents not only reduce blood pressure but also offer direct renoprotective effects. Felodipine has been shown to improve renal hemodynamics and reduce nephrosclerosis beyond its blood pressure effect (20), which is particularly beneficial in ischemic nephropathy. By reducing efferent arteriolar resistance (in the case of Losartan) and dilating afferent arterioles (Felodipine), we aimed to enhance glomerular perfusion and mitigate ischemia. The patient’s stable creatinine and controlled proteinuria at 13 months suggest that this combined approach was effective.

Compared to AA amyloidosis, which generally occurs after prolonged, uncontrolled systemic inflammation, the relatively early onset of FPGN and ischemic nephropathy in our patient underscores the need for vigilant monitoring of renal function in AOSD patients, even at early stages of the disease. Early identification and intervention are crucial for preventing irreversible renal damage (21, 22). This case contributes significantly to the understanding of renal complications in AOSD by emphasizing that the spectrum of renal pathology can extend beyond what has been previously documented in the literature. The coexistence of FPGN and ischemic nephropathy, both of which are rare in AOSD, suggests a need for heightened awareness among clinicians. The role of renal biopsy is essential to diagnosing specific renal pathologies and guiding treatment, ultimately improving patient outcomes.

## Conclusion

In conclusion, AOSD can present with unusual renal complications such as FPGN and ischemic nephropathy. This case highlights the importance of recognizing renal involvement in AOSD and tailoring treatment accordingly. A multidisciplinary approach involving rheumatologists, nephrologists, and pathologists is crucial for optimizing care and prognosis in patients with AOSD and renal involvement.

## Data Availability

The original contributions presented in the study are included in the article/supplementary material, further inquiries can be directed to the corresponding author.

## References

[1] Bywaters EG. Still’s disease in the adult. Ann Rheum Dis. (1971) 30:121–33. doi: 10.1136/ard.30.2.121, PMID: 5315135 PMC1005739

[2] Yoshida S Koga T Fujita Y Yatsuhashi H Matsumoto H Sumichika Y. Serum mac-2 binding protein glycosylation isomer and galectin-3 levels in adult-onset still’s disease and their association with cytokines. Front Immunol. (2024) 15:1385654. doi: 10.3389/fimmu.2024.1385654, PMID: 38711500 PMC11073344

[3] Arya PVA Marnet E Rondla M Tan JW Unnikrishnan D Buller G. Renal manifestations in adult-onset still’s disease: a systematic review. Rheumatol Int. (2024) 44:1209–18. doi: 10.1007/s00296-024-05578-5, PMID: 38625385

[4] Rivera F Gil CM Gil MT Batlle-Gualda E Trigueros M Olivares J. Vascular renal AA amyloidosis in adult still’s disease. Nephrol Dial Transplant. (1997) 12:1714–6. doi: 10.1093/ndt/12.8.1714, PMID: 9269657

[5] Serratrice J Granel B Disdier P Weiller PJ Dussol B. Resolution with etanercept of nephrotic syndrome due to renal AA amyloidosis in adult still’s disease. Am J Med. (2003) 115:589–90. doi: 10.1016/j.amjmed.2003.04.001, PMID: 14599647

[6] Domingues RB da Gama AM Caser EB Musso C Santos MC. Disseminated cerebral thrombotic microangiopathy in a patient with adult’s still disease. Arq Neuropsiquiatr. (2003) 61:259–61. doi: 10.1590/s0004-282x2003000200018, PMID: 12806507

[7] Quemeneur T Noel LH Kyndt X Droz D Fleury D Binaut R. Thrombotic microangiopathy in adult still’s disease. Scand J Rheumatol. (2005) 34:399–403. doi: 10.1080/03009740510026689, PMID: 16234190

[8] Salamon L Salamon T Morovic-Vergles J. Thrombotic microangiopathy in adult-onset still’s disease: case report and review of the literature. Wien Klin Wochenschr. (2009) 121:583–8. doi: 10.1007/s00508-009-1217-4, PMID: 19890748

[9] Bennett AN Peterson P Sangle S Hangartner R Abbs IC Hughes GR. Adult onset still’s disease and collapsing glomerulopathy: successful treatment with intravenous immunoglobulins and mycophenolate mofetil. Rheumatology (Oxford). (2004) 43:795–9. doi: 10.1093/rheumatology/keh172, PMID: 15039497

[10] Kumar S Sheaff M Yaqoob M. Collapsing glomerulopathy in adult still’s disease. Am J Kidney Dis. (2004) 43:e19.1–7. doi: 10.1053/j.ajkd.2003.11.025, PMID: 15112192

[11] Kang JH. IgA nephropathy in adult-onset still’s disease after tocilizumab treatment: a case report. Int Urol Nephrol. (2022) 54:1167–8. doi: 10.1007/s11255-021-02956-x, PMID: 34269969

[12] Takizawa Y Kanda H Sato K Kawahata K Yamaguchi A Uozaki H. Polymyositis associated with focal mesangial proliferative glomerulonephritis with depositions of immune complexes. Clin Rheumatol. (2007) 26:792–6. doi: 10.1007/s10067-006-0200-y, PMID: 16541204

[13] Wu Q Tanaka H Hirukawa T Endoh M Fukagawa M. Characterization and quantification of proliferating cell patterns in endocapillary proliferation. Nephrol Dial Transplant. (2012) 27:3234–41. doi: 10.1093/ndt/gfr797, PMID: 22431704

[14] Stangou M Bantis C Skoularopoulou M Korelidou L Kouloukouriotou D Scina M. Th1, Th2 and Treg/T17 cytokines in two types of proliferative glomerulonephritis. Indian J Nephrol. (2016) 26:159–66. doi: 10.4103/0971-4065.159303, PMID: 27194829 PMC4862260

[15] Kadavath S Efthimiou P. Adult-onset still’s disease-pathogenesis, clinical manifestations, and new treatment options. Ann Med. (2015) 47:6–14. doi: 10.3109/07853890.2014.971052, PMID: 25613167

[16] Ananthaneni A Shimkus G Weis F Adu-Dapaah E Lakra R Ramadas P. Adult-onset still’s disease with concurrent thrombotic microangiopathy: observations from pooled analysis for an uncommon finding. Eur J Haematol. (2024) 112:484–92. doi: 10.1111/ejh.14142, PMID: 37997494

[17] Simeoni M Borrelli S Garofalo C Fuiano G Esposito C Comi A. Atherosclerotic-nephropathy: an updated narrative review. J Nephrol. (2021) 34:125–36. doi: 10.1007/s40620-020-00733-0, PMID: 32270411

[18] Nankivell BJ Borrows RJ Fung CL O’Connell PJ Allen RD Chapman JR. The natural history of chronic allograft nephropathy. N Engl J Med. (2003) 349:2326–33. doi: 10.1056/NEJMoa020009, PMID: 14668458

[19] Feng J Meir L Ghaw O. Canakinumab and mycophenolate mofetil in managing proteinuria/renal amyloidosis secondary to adult-onset still’s disease. Rheumatol Adv Pract. (2023) 7:rkad046. doi: 10.1093/rap/rkad046, PMID: 37207268 PMC10188300

[20] Francischetti A Ono H Frohlich ED. Renoprotective effects of felodipine and/or enalapril in spontaneously hypertensive rats with and without L-NAME. Hypertension. (1998) 31:795–801. doi: 10.1161/01.hyp.31.3.795, PMID: 9495263

[21] Delplanque M Pouchot J Ducharme-Benard S Fautrel BJ Benyamine A Daniel L. AA amyloidosis secondary to adult onset still’s disease: about 19 cases. Semin Arthritis Rheum. (2020) 50:156–65. doi: 10.1016/j.semarthrit.2019.08.005, PMID: 31488308

[22] Blank N Schonland SO. Chronic inflammation and AA amyloidosis. Dtsch Med Wochenschr. (2013) 138:1835–8. doi: 10.1055/s-0033-1349428 24006165

